# The Potential Role of Astrocytes in Parkinson’s Disease (PD)

**DOI:** 10.3390/medsci8010007

**Published:** 2020-01-27

**Authors:** Hiluf Hindeya Gebreyesus, Teklu Gebrehiwot Gebremichael

**Affiliations:** 1 School of Medicine, Biomedical Sciences, College of Health Sciences, Mekelle University, P.O. Box: 1871 Mekelle, Tigray, Ethiopia; 2 School of Pharmacy, College of Health Sciences, Mekelle University, P.O. Box: 1871 Mekelle, Tigray, Ethiopia; tekluu2020@gmail.com

**Keywords:** neuron-astrocyte interactions, glutamate clearance, EAATs, EAAT2/GLT1, APQ4, PARK1-9 genetic mutations, Parkinson’s diseases

## Abstract

Astrocytes are multi-functional cells, now recognized as critical participants in many brain functions. They play a critical physiological role in the clearance of neurotransmitters, such as glutamate and gamma-aminobutyric acid (GABA), and in the regulation of K^+^ from the space of synaptic clefts. Astrocytes also express the excitatory amino acid transporters (EAATs) and aquaporin-4 (AQP4) water channel, which are involved in both physiological functions and neurodegenerative diseases (ND). Some of the ND are the Alzheimer’s (AD), Huntington’s (HD), Parkinson’s diseases (PD), Cerebral edema, amyotrophic lateral sclerosis (ALS), and epilepsy pathological conditions in specific regions of the CNS. Parkinson’s disease is the second most common age-related neurodegenerative disorder, characterized by degeneration of dopaminergic neurons of the substantia nigra pars compacta (SNpc). These project to the striatum, forming an important pathway within the basal ganglia. Mostly, PD has no clear etiology, and the mechanism of dopaminergic (DA) neuron loss is not well illustrated. The results of various studies suggest that astrocytes are involved in the pathophysiology of PD. Evidence has shown that the down-regulation of EAAT-2/GLT-1 and AQP4 expression is associated with PD pathogenesis. However, controversial results were reported in different experimental studies about the expression and function of EAAT-2/GLT-1 and AQP4, as well as their colocalization in different brain regions, and their involvement in PD development. Therefore, under neurological disorders, Parkinson’s disease is related to the genetic and phenotypic change of astrocytes’ biology. In this review, the authors summarized recent their research findings, which revealed the involvement of EAAT-2/GLT-1 and AQP4 expression, the physical interaction between EAAT-2/GLT-1 and AQP4 in astrocyte function, and their potential role in the development of PD in SNpc and Subthalamic nucleus (STN) of the basal ganglia nuclei.

## 1. Introduction

Astrocytes contribute to the homeostasis and regulation of the extracellular level of glutamate, GABA, and adenosine fundamental neurotransmitters in the CNS. The multi-functional role of astrocytes, includes their ability to uptake, release, and metabolize these neurotransmitters [1,2]. In this review, we concentrated on the role of astrocytes in the uptake of glutamate neurotransmitter, and their role in PD pathogenesis. Study results have been suggested that astrocytes may be involved in the function and pathophysiology of PD development, in the substantia nigra pars compacta (SNpc) neuro-inflammation and a key role as sensors of brain homeostasis [3,4,5]. Parkinson’s disease (PD) is the second most common age-related motor neurodegenerative disorder, and pathologically characterized by progressive degeneration of dopaminergic (DA) neurons in the SNpc [3,5,6] and STN [6]. Most PD etiology, and the mechanisms of DA neuronal degradation, have not yet been understood. However, mitochondrial damage, energy failure, oxidative stress, excitotoxicity, neuro-inflammation, and the impairment of signal transmission pathways may be involved in PD pathogenesis [3,5,6]. The structural and functional distribution of excitatory amino acid transporters (EAAT1-5) in astrocytes involves a function in glutamate clearance. Glutamate uptake from the synaptic cleft is mediated by astrocytes, mainly due to the presence of high-affinity glutamate transporter, EAAT1 and predominantly EAAT2 in the human brain [4,7]. 

Aquaporins (AQPs) are a small and very hydrophobic family of water channel membrane proteins, that control water homeostasis. Many AQP family members have been identified in a wide range of organisms. However, in this review, we will focus on the physiological role of aquaporin-4 (AQP4) subtype in astrocytes. The AQP4 water channel is a protein marker, mainly found on the membrane of perivascular astrocytes end-feet processes, responsible for brain water homeostasis [7]. The two astrocyte protein markers, the AQP4 channel and the glial fibrillary acidic protein (GFAP), have also been implicated in several physiological and pathological conditions in the central nervous system (CNS), as well as the blood–brain barrier (BBB) [8]. The immunofluorescence analysis of substantia nigra (SN) revealed that 1-methyl-4-phenyl 1,2,3,6-tetrahydropyridine (MPTP) injections led to the strong up-regulation of AQP4 and GFAP, both perivascularly and in non-end-foot membranes of reactive astrogliosis, compared to the saline treated animals [7,9]. 

A loss of astrocyte polarization may compromise the ability of astrocytes to regulate volume and water transport in the CNS. AQP4 also functionally cooperates with Kir4.1 to regulate water and K^+^ exchange at these sites [7]. EAAT-2 and AQP4 are functionally linked on the surface of astrocytic membranes’ macromolecular complex [10]. Therefore, this suggests involvement of AQP4 in PD, and may explain the reason PD patients exhibit water accumulation in SN. Hence, study data show that, decreased EAAT2 and AQP4 expression and function, have been associated with the PD pathophysiology [3,4,7]. Up-regulation and down-regulation of the mis-localization of the perivascular pool of AQP4 is implicated in several different brain disorders [4].

## 2. The Physiological Role of Astrocytes 

The brain, composed of neurons and glial cells, has high-order functions. Astrocytes are the most numerous, star-shaped, and highly heterogeneous in their morphology, as well as a multi-functional subtype of the four glial cells located in the CNS. They are involved in the maintenance of the health and function of the human CNS [5,11,12,13]. Astrocytes are highly polarized cells with membrane domains and strategically located in the brain, between the neurons and the brain’s blood vessels (perivascular end-foot processes) [4]. A recent experimental study strongly suggests the dynamic specialized functional unit of astrocytes and their link with neurons [12]. The astrocyte peri-synaptic membranes express glutamate transporters, Kir4.1 potassium channels, Na^+^/K^+^-ATPase, Na-K-Cl cotransporter (NKCC1), as well as AQP4 channels [4]. In electrophysiology, astrocytes are considered to be electrically non-excitable cells, with a constant resting membrane potential (−90mV) of specific physiological response to electrical stimulation [12,13]. 

The trophic and structural role of astrocytes also contribute to the dynamic components of brain connectivity and function [11]. Astrocytes perform several important physiological functions, including physical and metabolic support of neurons, repairing neurons, ion and water homeostasis, chemical signal transmission, regulation of energy metabolism and neurogenesis, blood flow regulation, immune and oxidative stress defense, detoxification, acid-base balance, and reaction to injury in the brain [5,11,12,14,15]. They also contribute to the formation and maturation of synapses and receptor trafficking [12]. Moreover, they are actively involved in the regulation of the extracellular space volume, communication pathways, neuroprotection, synaptic plasticity [11,12,16], central chemoception, and the regulation of sleep [2]. These properties of astrocytes allow them to coordinate the activity of the whole neuronal pool [12].

## 3. Neuron-Astrocyte Interactions in the Brain

Neuron and astrocyte interactions are complex, and both are coupled together by gap junction connexins [15]. Astrocytes are also connected with other nearby astrocytes, with the help of gap junctions [17]. Neurons are critically dependent on the intrinsic protective and supportive properties of astrocytes. The astrocyte-neuron relationship begins during development, when astrocytes regulate neurogenesis by guiding and supporting neuronal migration, survival, and process extension [7,18]. Therefore, astrocytes are highly recognized to play important physiological roles in neuronal development, neurotransmission, synaptic plasticity, and maintenance of brain homeostasis. The interactions between neurons and astrocytes are crucial for chemical signaling, energy metabolism, extracellular ion homeostasis, volume regulation, and neuro-protection in the CNS [1,7,18]. Likewise, astrocytes are the source of glucose, oxygen, metabolites, such as lactate, fatty acids, as well as trophic factors that sustain neuronal health [19].

Astrocytes are involved in the uptake and release of neurotransmitters, trophic factors and energy substrates for neurons, and in the control of water and ion homeostasis [7]. Astrocytes produce and release different trophic factors to regulate neurogenesis processes, synapse formation, maintenance, and remodeling. Some of them are the brain-derived neurotrophic factor (BDNF), glial cell-derived neurotrophic factor (GDNF), nerve growth factor (NGF), insulin-like growth factor (IGF), and thrombospondin [7,17]. Bi-directional communication between neurons and astrocytes at the synapses also have a critical role in regulating chemical neurotransmission. Moreover, astrocytes possess functional receptors for the uptake of neurotransmitters, and themselves respond to neurotransmitter stimulation by releasing transmitter molecules. In the CNS, the uptake of the neurotransmitter, glutamate, from the synaptic cleft by astrocytes occurs through Na^+^-dependent excitatory amino acid transporters (EAATs) [20,21]. In humans, EAATs provide the Na^+^-mediated driving force for glutamate uptake from the extracellular synaptic space [7,22]. Astrocytes are also known for activating ionotropic and metabotropic glutamate receptors, which, in turn, lead to Ca^2+^ influx during synaptic activity [17]. Glutamate is then converted into glutamine by glutamine synthetase (GS), and is released back to the pre-synaptic terminal of neurons, where it is converted to glutamate by the enzyme, glutaminase [7,20,22].

Regarding functionally specialized cellular and molecular settings, astrocytes are highly heterogeneous in morphological appearance that express a mass of receptors, channels, and membrane transporters [7,14]. Therefore, astrocytes, from different brain regions, express different sets of proteins that are responsible for glutamate clearance from the extracellular synaptic cleft [3,20,23]. Currently, there are five families of membrane proteins of EAAT, that have been discovered in the regional and cellular distribution of the human brain [3,20,23]. These glutamate transporters, known as *EAAT1* in human (*GLAST* in rodents), *EAAT2* in humans (*GLT-1* in rodents), *EAAT3* in humans (*EAAC1* in rodents), *EAAT4,* and *EAAT5*, share a common nomenclature in both human and rodents [3,20,22,23,24,25]. The human genes for the five EAAT glutamate transporter sub-types of soluble carrier families are also different, named, *SLC1A3, SLC1A2, SLC1A1, SLC1A6, and SLC1A7,* respectively [24]. 

The EAAT1 and EAAT2 are predominantly found in the astrocytes of the human brain [3,20,22,23]. The EAAT1 and EAAT2 localization has also been identified in neuronal regions [20]. The EAAT1/GLAST immune-staining protein expression is restricted to glial cells and is the most abundant of the EAATs in the cerebellum and retina [3,23,26], and moderately in the hippocampus and forebrain [3,26]. EAAT2 is the most abundant EAAT in the forebrain [20,26], cerebral cortex, and hippocampus [3], spinal cord, and with minor expression in the cerebellum [26]. Almost all the EAAT2 are expressed in astroglial cells in the normal brain and spinal cord [23], which is most strongly expressed in the forebrain [27]. The EAAT3 is widely found in peripheral tissues [27], as well as in both neurons and astrocytes [23,27], cortex, hippocampus, and striatum [20]. EAAT4 is highly enriched in the Purkinje cells of the cerebellum [20,23], but limited in the forebrain, and EAAT5 is expressed primarily in rods of photoreceptors and bipolar cells of the retina [3,20,23] and amacrine cells [3]. In the brain, EAAT3 expression is highly expressed in the hippocampus of young adult rat [3,28] and cortex [28], and with lower levels in the midbrain, striatum, and cerebellum [28]. A recent study also showed that EAAT4 and EAAT5 are expressed in type I and II vestibular hair cells [3].

Study reports, regarding functional heterogeneity of the cellular and molecular membrane markers, have been implicated in the pathogenesis of astrocytic-mediated disease processes [7,29,30]. Neurodegenerative diseases and glial pathophysiology are now becoming a sound point for neurological studies. Hence, astrocytic membrane protein dysfunction has been extensively observed in the pathogenesis of numerous neurodegenerative conditions [7,14]. This is mainly associated with the transport mechanisms of the EAAT transporters’ (EAAT1-5) performance, location, and distribution in the human brain. Current evidence has stressed the link between astrocytes and the pathological conditions of several neurodegenerative diseases (ND) in the human CNS, such as Alzheimer’s disease (AD), Huntington’s disease (HD), Parkinson’s diseases (PD), cerebral edema, amyotrophic lateral sclerosis (ALS), epilepsy [12,13,14,28], stroke, inflammatory diseases, migraines, depression [12,13], and Drug addiction [27]. Therefore, an in-depth understanding of the fundamental role of astrocytes is essential to understand the mechanisms of chronic neurodegenerative diseases in CNS pathological conditions, such as PD. In this review, we will focus on the role of astrocytes dysfunction in PD neuronal disorder.

## 4. The Role of Astrocytes in Parkinson’s Disease 

### 4.1. Excitotoxicity and Parkinson’s Disease 

Parkinson’s disease (PD) is the second most common motor neurodegenerative disease, after Alzheimer’s disease (AD) [12,31]. PD results primarily from the progressive, selective, and irreversible massive loss of dopaminergic (DA) neurons in the SNpc [4,12,32,33], and the formation of Lewy bodies [3,32,33]. It is most common in older aged individuals, and characterized by a decrease or loss of movement, rigidity, rest tremor, and bradykinesia of impaired motor symptoms [32,34,35]. Pathologically, these symptoms are associated with the loss of dopaminergic neurons in the SNpc [5,32,35]. Therefore, the protection of substantia nigral dopamine neurons from progressive degenerative death, and cell replacement of novel dopamine neurons, are hopeful strategies against PD in humans [5,33]. 

The basal ganglia nuclei process the inputs flowing from the cortex to produce an output signal that returns through the motor thalamus to the cortex, in order to modulate movement performance. The basal ganglia micro-circuit consists of four nuclei: Corpus striatum (or striatum), globus pallidus, substantia nigra, and subthalamic nucleus [36]. Recent studies have shown that the sub-thalamic nucleus receives direct excitatory projections from the primary motor cortex [36], and dopaminergic projections from the SNpc [36,37] and striatum [38]. Therefore, the degeneration of dopaminergic neurons in the substantia nigra (SN) influences the direct, and indirect, pathway in the basal ganglia microcircuits [35].

A reduction in glutamate uptake activity may result in an excess glutamate accumulation in the synaptic cleft, which over-stimulates the inotropic and metabotropic glutamate receptors in the postsynaptic membranes, thereby leading to neuronal excitotoxicity [3,5]. This neuronal excitotoxicity is a known cause of neurodegenerative disorders [5]. The successive over-stimulation of glutamatergic N-methyl-D-asparate (NMDA) receptor induces numerous neurotoxic effects, such as intracellular Ca^2+^ homeostasis dysfunction, increase nitric oxide (NO) and reactive oxygen species production, as well as reactive nitrogen radicals [3,21], resulting in mitochondrial dysfunction [3], the activation of proteases, and an increase in cytotoxic transcription factors [21]. Therefore, glutamate excitotoxicity can induce dopaminergic neuron death, movement disorder, cognitive impairment, and pathogenesis of PD [3]. The malfunction of glutamate metabolism and other factors could also be involved in PD genesis, through different pathways and neuronal excitotoxicity [3,5]. Astrocytic EAATs dysfunction has been associated with the pathogenesis of numerous neurological disorders. At high extracellular glutamate concentrations, glutamate can induce neuronal cell death by a complex mechanism, termed excitotoxicity [3,39]. 

### 4.2. Astrocytes and Parkinson’s Disease 

The cause of PD is not always due to the loss of these dopaminergic neurons and continuous depletion of dopamine, but the concept that it could be attributed to astrocytes, resulting in neuropathological or neuroprotective functions, is becoming gradually more recognized [37]. Dopaminergic (DA) neuron depletion of variable degrees is more severe in the basal ganglia subdivisions of the Globus pallidus external (GPe), than the globus pallidus internal (GPi) in PD [40]. However, neurons are not the only cell types in the GPe that may experience variations in disease pathophysiology. In vivo studies have determined that the autonomous activity of GPe neurons is believed to contribute to basal ganglia neural microcircuits dysfunction in PD [35,41]. On the other hand, Eun-Hye Joe, et al. [41], have reviewed the three classes of glial cells in the brain, named oligodendrocytes, microglia, and astrocytes, which are also involved in GPe pathophysiology states. 

The relative abundance of astrocytes in GPe indicates that they have an important role in regulating GPe function [31,41]. Such studies, at the cellular level, reveal important features of the organization and synaptic dynamics of GPe circuits, which make them subject to dysfunction in PD [35,41]. However, the role of astrocytes in PD is poorly understood. From pathological experimental studies, an increase in the number of astrocytes, as well as in glial fibrillary acidic protein (GFAP) expression, is observed in PD pathogenesis. Recent data show that the potential causes of nigral cell (SNc) degeneration in PD is the involvement of mitochondrial defects, elevated oxidative stress, and glutamatergic stimulation [37,41]. 

Astrocytes produce many neurotrophic molecules (BDNF, GDNF, and IGF) and growth factors, that are important in the development and survival of dopaminergic neurons [3,42] and glial cells. Their action against toxins and reactive oxygen species (ROS) is significant in the treatment of PD and BBB disruption [42]. The GDNF family comprises ligands, such as GDNF, Neurturin (NRTN), artemin (ARTN), and persephin. Therefore, this GDNF, secreted by astrocytes and pericytes, is vital for the existence of dopaminergic neurons, peripheral motor neurons, and neurons from the locus coeruleus [3,42]. Astrocytes respond to various chronic neurodegenerative diseases, such as in AD, PD, ALS, and other disorders [7,37]. 

In animal models, the most two neuro-toxicants, commonly used to induces pathology and PD, are 1-methyl-4-phenyl 1, 2, 3, 6-tetrahydropyridine (MPTP) and 6-hydroxydopamine (6-OHDA). The management of neurotoxins, such as MPTP and (6-OHDA), promote the degeneration of dopaminergic neurons in the nigrostriatal pathway [43]. MPTP is systemically administrated to mice models and, because of its highly lipophilic property, it easily crosses the blood-brain-barrier, where it binds mainly to astrocyte lysosomes [43,44]. Therefore, a study conducted by Meredith and Rademacher [43] reported that neuro-degeneration in SNpc, following MPTP treatment, was carried out on the C57BL/6 mice. So that, in MPTP models, MPTP metabolism via astrocytes monoamine oxidase-B (MAO-B) of MPTP produces active MPP+ metabolite within astrocytes [34,45]. Research studies have also shown astrocytic neuro-protection in the course of exposure to MPTP. MPP+ accumulation in dopaminergic neurons induced neurotoxicity, primarily by inhibiting complex I of the mitochondrial electron transport chain processes [34]. Such protection is determined by the increased GFAP immune-reactivity of the astrocytes protein marker in the striatum [34]. Besides, in the 6-OHDA rat models of PD, 24 h after SNc lesion, the GFAP levels increased and peaked almost four times high, at four days after injection [45]. 

#### 4.2.1. EAAT1 and EAAT2 Dysfunction in Astrocytes and Parkinson’s Disease 

Glutamate is the predominant excitatory neurotransmitter in the human brain, and is regulated by the Excitatory Amino Acid Transporter (EAAT) subtypes, localized to neurons and astroglia cells [20,28]. EAAT1 and EAAT2 are highly specific glutamate transporters, that are expressed in astrocytes. Under physiological conditions, the main cellular source of EAAT1 and EAAT-2 are glial cells, especially astrocytes in the CNS [30]. EAAT1 and EAAT2 may mediate glutamate clearance from the synaptic cleft to prevent potential neuronal excitotoxicity and hyper-excitability [3,7,11,22]. It is confirmed that the glutamate transporter, EAAT1, is more strongly expressed at glial membranes facing the neuropil than in those facing vessels or pia [46]. Considering that EAAT1 plays a lesser important role in the glutamate uptake, decreased EAAT1 expression is possibly a product in the pathogenesis of PD. GLAST-mediated reuptake component in the striatum may be relatively minor. Moreover, GLAST expression is also shown to increase in the striatum in 6-OHDA lesion rats [3], as astrocytic GLT-1 is mainly responsible for glutamate reuptake. These studies reveal that GLAST may play a compensatory role when GLT-1 function is compromised in PD.

The EAAT2 subtype is functionally the main regulator of extracellular glutamate levels in most brain regions [47]. EAAT2/GLT-1 is widely distributed in the CNS, and is mainly expressed in astrocytes in the forebrain, cerebral cortex, hippocampus, and other regions [3,48]. The EAAT2 transporters are the most abundant, and absorb glutamate from the synaptic cleft, being responsible for up to 95% of its total uptake [49]. However, astrocyte dysfunction may lead to numerous neurodegenerative diseases [7]. EAAT2 dysfunction leads to extracellular glutamate accumulation and is associated with many neurodegenerative diseases, like Huntington’s disease [49], Alzheimer’s disease (AD), Parkinson’s disease (PD) and amyotrophic multiple sclerosis (AMS) [39,49]. EAAT2 dysfunction is also expressed in several psychiatric diseases, such as schizophrenia, epilepsy, depression, and autism [39]. 

#### 4.2.2. AQP4 Dysfunction in Astrocytes and Parkinson’s Disease 

Astrocytes establish bi-directional communication with BBB components. Astrocytes also form contact with neighboring astrocytes via connexins of gap junctional channels [7]. There are two astrocyte protein markers, aquaporin-4 (AQP4) water channel and the glial fibrillary acidic protein (GFAP), implicated in several physiological and pathological conditions in the CNS and BBB [8,50]. The regulation of water permeability across the BBB is fundamental to maintain brain homeostasis. Aquaporins (AQP) are a family of tetrameric integral transmembrane proteins that establish a water-conducting channel from one side of the plasma membrane [8], which is the main molecular target for factors regulating water homeostasis in the human body. From the different types of AQPs distribution, AQP1 and AQP4 are the main water channels found in the human brain, precisely localized to choroid plexus, and astrocytes foot processes, respectively [7]. AQP4 channels are the predominant aquaporin densely clustered along with astrocyte end-foot processes of the BBB [7,50,51,52]. AQP4 expression is higher in the perivascular end-foot processes neighboring onto blood vessels and facing pia surface, than the synaptic glial processes [4,51]. Another study has also indicated AQP4 expression in endothelial cells, but absent from neurons, oligodendrocytes, and microglia [4].

Based on the quantitative immuno-gold labeling data, the perivascular AQP4 pool differs in density and size across the regions and sub-regions of the brain [4]. The AQP4 and GFAP immune-reactivity of two astrocytes end-feet protein markers are co-localized among the neuron bodies of the granular and Purkinje layers in the cerebellum of neonate adult rats [8]. Also, results from the immunofluorescence labeling and electron microscope quantitative immuno-gold analysis, showed that the perivascular density of AQP4 in mice is higher in SNpc than in the neocortex [4,53]. From the immuno-gold analyses, the a-syntrophin is also the most important factor determining the expression of perivascular AQP4 pool at the brain–blood interface [53]. Recent experimental studies indicate that AQP4 in astrocytes regulates various biological functions of the CNS, such as maintaining CNS water balance, spatial buffering of extracellular potassium, calcium signal transduction, regulation of neurotransmission [8,47,50], formation of memory, synaptic plasticity, astrocyte-to-astrocyte cell communication [8,52], learning [52], development of the BBB, and adult brain neurogenesis. Recently, the expression and regulation of AQP4 have been studied to understand the roles of AQP4 in several pathophysiological conditions. Under neuro-pathological conditions, much evidence has suggested that AQP4 participates in the onset and progression of patho-physiological disorders, such as Parkinson disease [4,24], depression [24], neuro-myelitis optical [24], Alzheimer disease [4,50,52], ischemia [50], K^+^ spatial buffering system [4,50], epilepsy, cerebral edema [4,47,50,52], stroke [4], and drug addiction [54]. AQP4 expression pattern deficiency reduced the differential degeneration of mid-brain dopaminergic neurons in experimental Parkinson’s disease [4,23].

The down-regulation of the AQP4 mRNA was also seen in PD patients when compared with age-matched healthy controls [51]. Similarly, the down-regulation of AQP4 expression has enhanced the sensitivity of dopaminergic neurons to neurotoxicity through the modulation of astrocytic neurotrophic factors [51,55]. There is also evidence that changes in the number of GFAP positive cells are involved in PD [8,55].

#### 4.2.3. EAAT2-AQP4 Interactions in Astrocytes and Parkinson’s Disease

EAAT2 and AQP4 both existed in the astrocytic end-foot membrane as macro-molecular complexes [10,23], and both are also depleted from plasma membranes of cultured astrocytes, exposed to neuromyelitis optica immunoglobulin G (NMO-IgG) [10]. In the mammalian brain and spinal cord, AQP4 is co-localized with glutamate transporters of EAAT1 and EAAT2, as well as with Kir 4.1 in the astrocyte plasma membrane, which suggests that AQP4 could modulate K^+^ and glutamate homeostasis [56]. Functionally, AQP4 and EAAT2 exist in astrocytic membranes, and are physically linked in a macromolecular complex of several brain regions and spinal cord [10,23,57,58,59,60]. The immunohistochemical analysis of non-pathologic human CNS tissue of both, cortical and the spinal cord, reveal that EAAT2 is normally co-localized with AQP4 in gray matter astrocytes [10]. 

The co-localization of AQP4 and EAAT2 was diminished in striatal astrocytes of equilibrative nucleoside transporter 1 (ENT1) null mice compared with wild-type littermates [59,60]. In addition, it was also observed co-localization between AQP4 and GFAP expression [59,60] in the striatum of ENT1 null mice [60]. A study conducted by Hinson et al. [10] has also found physical interaction between AQP4 and GLT-1 in transfected HEK-293 cells lines expressed EAAT2 transcripts at similar levels. Several studies used the co-immunoprecipitation, Western blot and immunohistochemistry and have found region-specific co-localization between GLT-1 and AQP4 throughout the brain [10,23,61], such as in cerebellum of AQP4 knockout mice [56,58], hippocampus and cortex [23,58]. This was also supported by the finding that GLT-1 expression was reduced upon exposure to antibodies in neuro-myelitis optica, targeting AQP4 [10]. 

The immunocytochemical and immunoprecipitation data suggested that regions of AQP4 loss in neuromyelitis optica [NMO) spinal cord lesions are deficient in EAAT2 [10]. The reduction of GLAST expression was also found in an AQP4 knock-out mice model [58]. A recent study reported that astrocytes lacking AQP4 express a reduced level of the astrocytic glutamate transport through EAAT2 [10]. The concentration of AQP4 protein in the plasma membrane and glutamate transporter are both reduced by exposure of primary astrocytes to NMO-IgG [10]. AQP4 deficiency increased glutamate levels in the synaptic cleft, which induced neuro-excitation activity in the hippocampus of AQP4 KO mice [50,58,62]. Also, the concomitant loss of both, EAAT2 and AQP4, reasonably explains the reduced glutamate transport, observed in cultured astrocytes exposed to NMO-IgG [10]. In contrast, another study found no physical interaction between the immune-expression patterns of AQP4 and GLT-1, in both CD1 and C57BL/6 adult mice [23]. Immuno-gold labeling and electron microscopy studies revealed that the astrocytic end-foot membrane contains dense AQP4 channel expression, which is co-localized with and Kir4.1 potassium channels. Recently, some studies suggested that, astrocyte AQP4 expression is functionally and mechanically in cooperation with GLT-1 [10], Kir4.1 to regulate water and K^+^ exchange [1,4,7,50,62]. 

#### 4.2.4. Genetic Studies in Astrocytes and Parkinson’s Disease

The dysregulation of EAAT1 and EAAT2 expression and function occurs at multiple levels, from abnormal genetic coding to altered post-translational modifications. Genetic dysregulation of EAAT2, such as single nucleotide polymorphisms (SNPs) and aberrant mRNA splicing of EAAT2, are known to impair EAAT2 expression and function [22]. The reduction of EAAT2 expression is associated with several neurological disorders, including amyotrophic lateral sclerosis (ALS), Alzheimer’s disease (AD), Parkinson’s disease (PD), schizophrenia, and epilepsy [35]. Several pharmacological treatments of astrocytes with ceftriaxone [26,63], estrogen [63,64], tamoxifen [63,64] and raloxifene [63] increase EAAT1 and EAAT2 expression at the transcription level, through the activation of nuclear factor-kappa light chain enhancer of activated B cells (NF-κB) pathway [26,63,64,65]. The role of nuclear factor-kB (NF-κB) on epidermal growth factor (EGF) and transforming growth factor-α (TGF-α) induces EAAT2/GLT-1 promoter activation and regulates its genetic transcriptional levels [26,63]. 

On the other hand, astrocytes treated with Manganese, for 6 h, decreased the GLAST protein expression, while estrogen and tamoxifen treated for 24 h increased GLAST protein expression [64]. The negative regulatory mechanisms of EAAT1 and EAAT2 promoter activity have been linked to the transcription factor yin yang 1 (YY1) [22,64,65]. Manganese and tumor necrosis factor-α (TNF-α) also decreased EAAT2 via activation of YY1 [22].

Therefore, recent genetic studies suggest that the death of dopaminergic neurons of the SNpc and striatum is the pathological hallmark of PD [3,5]. The exact cause of PD is unknown, however, about 50% of the PD patients are associated with a family history of genetic mutations expressed in astrocytes [5]. A current study, comparing the transcriptome of different human and mouse brain cell subtypes, demonstrated that many of the monogenic mutations of genes have been identified to be higher in astrocytes than neurons [5]. There are several well-known cell-specific genes, related to the functions of astrocytic biology. As mentioned in Table 1 below, the mutations of these genes are involved in the development of autosomal recessive PD pathogenic pattern, namely PARK1 (α-Synuclein), PARK2 (Parkin), PARK6 (PINK1 or PTEN-induced putative kinase-1), PARK7 (DJ-1), PARK8 (LRRK2 or Leucine-rich repeat kinase 2) [5,32,66], PARK8 (LRRK2) [5,32,33], NR4A2 (NURR1) [67], FBX07 (PARK15, F-box only protein 7], and vacuolar protein sorting (VPS35) [66,68]. Proteins encoded by these genes have been shown to have a role in astrocyte biology and PD pathogenesis. 

The evidence for PD-related monogenic mutations, having a role in astrocyte biology, has been mostly found in relation to α-Synuclein (PARK1) parkin (PARK2), DJ-1 protein (PARK7) [5,32,66,68], and PINK1 [5,68,73] gene expressions (Table 1). For instance, the accumulation of α-Synuclein-positive cytoplasmic inclusions (encoded by SNCA), in substantia nigra neurons and astrocytes, is the main histopathological feature of the PD [5]. DJ-1 protein expression may be involved in the modulation of nigrostriatal dopaminergic neuronal function [66]. Parkin mutation is also the second gene, identified in mitochondria, that causes early onset of Parkinsonism [32,68]. The expressions of PARK7 is higher in astrocytes than neurons in the human brain, and to be up-regulated in reactive astrocytes in patients with PD pathogenesis [5,32,68]. Hence, PARK7 Knockout (KO) and mutant astrocytes biology have been found to exhibit impaired glutamate uptake, by decreasing EAAT2 protein expression [5,68]. 

## 5. Conclusions

Glutamate and water homeostasis are physiologically crucial in maintaining the health of the human brain. Any alterations in the glutamatergic neurotransmission, and glutamate receptor function in astrocytes, may be involved in the neurodegenerative process and the development of the PD. Some research studies showed the physical interaction between EAAT2/GLT1 and AQP4 protein expression in astrocytes. AQP4 are co-localized with EAAT2/GLT-1 in nigrostriatal neurons. Therefore, the malfunction of the Astrocytic EAAT2 and AQP4 are involved in the pathogenesis of PD. The functions and biological features of astrocytes are altered by AQP4 gene deletion. This is because of the physical interactions, AQP4 Knockout down-regulated EAAT-2/GLT-1 protein expression and decreased the glutamate transporter mechanism in the synaptic cleft. Moreover, the EAAT2 and AQP4 gene mutations caused severe dopaminergic neuronal loss. The genetic mutations implicated in PD are more significantly expressed by α-Synuclein, Parkin, PINK1, DJ-1 FBXO7, and GBA genes in human astrocytes biology, during the PD pathogenesis.

## Figures and Tables

**Table 1:** Genes associated with Parkinson’s disease.

Gene Loci	Chromosomes	Proteins	Forms of PD and Age Onset	References
PARK1	4q21	α-Synuclein (SNCA)	Autosomal dominant, early onset	[5,6,33,69]
PARK2	6q25-27	Parkin	Autosomal recessive, early onset	[5,6,33,69]
PARK3	Unknown	2p13	Autosomal dominant,	[6,33,69]
PARK4	4q21	SNCA	Autosomal dominant, early onset	[6,33,66,69]
PARK5	4p14	UCH-L1	Autosomal dominant, idiopathic	[5,6,33,69]
PARK 6	p35–p36	PINK1	Autosomal recessive, early onset	[5,6,33,69]
PARK7	1p36	DJ-1	Autosomal recessive, early onset	[5,6,33,69]
PARK 8	12q12	LRRK2	Autosomal dominant, idiopathic	[5,6,33,66,69]
PARK 9	1p36	ATP13A2	Kufor-Rakeb Syndrome, early onset	[6,69]
NR4A2	2q22-23	NURR1	Autosomal dominant, late onset	[67,69]
VPS35	16q11.2	D620N	Autosomal dominant, late onset	[5,66,69,70]
FBXO7	22q12.3	F-Box Protein 7	Autosomal-recessive, early onset	[5,66,71]
GBA	1q21-22	Glucocerebrosidase	Autosomal-recessive, late onset	[5,6,66,72]

## Abbreviations

PD	Parkinson’s disease
SNpc	Substantia Nigra pars compacta
EAATs	Excitatory amino acid transporters)
AQP4	Aquaporin-4
DA	dopaminergic neurons
ND	Neurodegenerative diseases
BBB	Blood–brain barrier
GLAST	glutamate aspartate transporter
GLT1	Glutamate transporter 1
SN	Substantia nigra
GPe	Globus pallidus external
GPi	Globus pallidus internal
MPTP	1-methyl-4-phenyl 1,2,3,6-tetrahydropyridine
6-OHDA	6-hydroxydopamine
NMO-IgG	Neuromyelitis Optica immunoglobulin G
HEK-293	Human embryonic kidney cells
